# Editorial: New insights into schizophrenia-related neural and behavioral phenotypes

**DOI:** 10.3389/fncel.2023.1202230

**Published:** 2023-05-10

**Authors:** Yuh-Man Sun, Ji Chen

**Affiliations:** ^1^Retired, London, United Kingdom; ^2^Department of Psychology and Behavioral Sciences, Zhejiang University, Hangzhou, China; ^3^Department of Psychiatry, The Fourth Affiliated Hospital, Zhejiang University School of Medicine, Yiwu, Zhejiang, China

**Keywords:** schizophrenia, excitation/inhibition (E/I) imbalance, extracellular vesicles (EVs), voltage-gated ion channels, (VIP)-expressing GABAergic neurons, human iPSC derived neurons, working memory (WM), Reelin

The etiology of schizophrenia (SCZ) is multifactorial and complex. Scientists employed animal models, human post-mortem tissue, imaging, bioinformatics, and recently human induced pluripotent stem cell (hiPSC)-based modeling to dissect the underlying multifaceted mechanisms of the disease. The emerging consensus is that cortical pathology is one of the fundamental features of schizophrenia (Selemon, 2001; Selemon and Zecevic, 2015; Parnanzone et al., 2017; Di Biase et al., 2019). The neocortex consists of around 80% of glutamatergic excitatory pyramidal neurons and 20% of GABAergic inhibitory interneurons (Harris and Shepherd, 2015; Lodato and Arlotta, 2015; Tatti et al., 2017; Musall et al., 2023). Mounting evidence suggests that aberrant connectivity of cortical macrocircuitry and microcircuitry plays a pivotal role in SCZ, especially excitation/inhibition (E/I) imbalance at the molecular, cellular, cell-type, and regional levels (Yizhar et al., 2011; Lisman, 2012; Marin, 2012; Gao and Penzes, 2015; Sohal and Rubenstein, 2019; Liu et al., 2021). The imbalance in excitatory and inhibitory information can cause disruption in sensory and working memory (Casanova et al., 2007; Opris and Casanova, 2014). The knowledge derived from the article collection in this Research Topic will be of help for understanding and unraveling the pathophysiology of SCZ under the framework of E/I imbalance.

At the molecular and cellular level, dysfunction of corticolimbic glutamatergic neurotransmission plays a critical role in the manifestations of schizophrenia (Coyle, 1996; Paz et al., 2008; Egerton et al., 2020). Glutamatergic neurons represent the primary excitatory afferent and efferent systems innervating the cortex, limbic regions (e.g., hippocampus and amygdala), and striatum (Coyle, 1996; Moghaddam, 2003). This orchestrates intricate interplays amongst neuronal networks (e.g., glutamatergic, GABAergic, dopaminergic, serotonergic neurotransmission, etc.). Dysfunction in one of those neuronal networks could alter an E/I balance (Belmer et al., 2016; Hayashi-Takagi, 2017; Sonnenschein et al., 2020). Let us now focus on the units of neuronal networks, i.e., neuronal synapses. It is well documented that synaptopathy underlies a variety of psychiatric disorders (Hayashi-Takagi, 2017; Obi-Nagata et al., 2019; Friston, 2023). Our study based on hiPSC modeling showed that an array of genes involving glutamatergic, GABAergic, dopaminergic, and cholinergic synapses are downregulated in the neurons derived from clozapine-responsive SCZ patients (e.g., *GRIN2A, GRM1, VGLUT3, VGLUT2, GNB2, ADCY1, ADCY2, ADCY5, ADRBK1, GABBR2, GABBR3, GAT1, VGAT, GAD1, GABARAPL2, DRD1, CAMK2A, CAMK2B, PPP2R2C, PPP2CB, PPP2R5B, MAOA, MAPK11, KIF5A; CHRM3, KCNQ2*). The majority of those genes are restored by clozapine, especially the function of NMDA receptors (Hribkova et al., 2022). It was also observed a significant reduction in VGLUT1/PSD95-positive synapses in SCZ neurons (Hribkova et al., 2022). PSD95 plays critical roles in maintaining the balance between excitatory and inhibitory synapses, synapse development, and synaptic plasticity (Zeng et al., 2016; Lambert et al., 2017; Smith et al., 2017). In this Research Topic, Chen et al. report that defective dendritic spines and autism-like behaviors observed in the Fragile X messenger ribonucleoprotein 1 (*Fmr1*) knockout mice are rescued by dihydrotestosterone (DHT), whereby DHT increases PSD95 expression by abating the Fragile X messenger ribonucleoprotein (Fmrp)-mediated miR-125a/RISC inhibition of PSD95 productions. Moreover, neuronal extracellular vesicles (EVs) are also a key player in neuronal synapses. CD63 is one of the EV proteins and facilitates vesicular trafficking through endosomal pathways. Hendricks et al. find that Tsp42Ee and Tsp42Eg (Tsps), CD63 homologs in *Drosophila*, influence the synaptic cytoskeleton and membrane composition by regulating Futsch loop formation and synaptic levels of SCAR and PI(4,5)P 2. Tsps influence the synaptic localization of several vesicle-associated proteins including Synapsin, Synaptotagmin, and Cysteine String Protein. In a review article, Jiao et al. delineate the roles of neuronal EVs in cellular homeostasis, intercellular communication, and phenotypic changes in the recipient cells via sophisticated machineries. Aberrant EVs cause neuropathy and lead to neurological disorders, which echoes the EVs' role in SCZ (Wang et al., 2022). Others and we also observed abnormalities in EVs in SCZ. A study reported that peripheral EVs in psychotic patients contain higher levels of proteins involving the regulation of glutamatergic synaptic plasticity (Tunset et al., 2020). Our study showed that some of the genes responsible for synaptic vesicle cycle (e.g., *VGLUT2, VGLUT3, VGAT, CPLX2, RAB3A, STX1B1, and ATP6V1A*) are down-regulated in clozapine-responsive SCZ neurons (Hribkova et al., 2022).

Furthermore, synaptic ion channels also play a pivotal role in shaping synaptic communication and plasticity (Voglis and Tavernarakis, 2006; Burke and Bender, 2019) and accumulating data suggest that polymorphisms and mutations in ion channels link to the susceptibility or pathogenesis of psychiatric diseases (Imbrici et al., 2013). Our study also reveals that a score of genes encoding ion channels [e.g., *SLC4A4, SLC32A1, SLC13A4, SLC1A4, SLC17A8, SLC17A6, SCN2A, ATP1B1, SCN3A, ATP1A2, ATP1A3, SLC6A1, HCN4, KCNK10, KCNB1(Kv2.1), KCNH8 (Kv12.1), KCTD2, ATP1B1, KCNQ2 (Kv7.2), ATP1A2, ATP1A3, TMEM38A, KCNG1 (Kv6.1), KCNF1 (Kv5.1), KCNJ4 (Kir2.3), CACNG5, and CACNG8*] are down-regulated in clozapine-responsive SCZ neurons, in which some are restored by clazopine (Hribkova et al., 2022). Those studies suggest that the dysregulation of ion channel genes is associated with SCZ. Understanding the role of individual channels in SCZ is an insurmountable task due to the numerous constellations of subtypes. Therefore, computing and mathematical modeling would be useful tools to explore the involvement of ion channels in SCZ and for drug testing. In this Research Topic, Rathour and Kaphzan employ neuronal modeling to compute how variability of voltage-gated ion channels (VGICs), including fast Na^+^, delayed rectifier K^+^, A-type K^+^, T-type Ca^++^, and HCN channels, affects information transfer in neurons. They show that the A-type K^+^ channel is the major regulator of information transfer. McGahan and Keener construct a novel mathematical model for heteromeric potassium channels that captures both α-subunit number and type present in each channel.

At the cell-type level, a review article by Apicella and Marchionni elucidate the role of vasoactive intestinal polypeptide (VIP)-expressing GABAergic neurons in the neocortical areas via disinhibitory and inhibitory effects on the intricate cortical circuits, which translates the external stimuli into underlying behaviors. The authors mentioned the effect of ErbB4 knockout mice on cortical microcircuits. ErbB4 ablation reduces the activity of VIP-expressing neurons also witnessed with an increase excitatory neuronal activity, suggesting a direct inhibitory effect of the VIP (Batista-Brito et al., 2017). ErbB4, a receptor of the schizophrenia-linked protein neuregulin-1, regulates glutamatergic synapse maturation, plasticity, NMDAR-mediated neurotransmission, and the migration of GABAergic interneurons (Flames et al., 2004; Li et al., 2007). The dopaminergic system plays a crucial role in the pathophysiology of schizophrenia (Collo et al., 2020; Martel and Gatti McArthur, 2020; Sonnenschein et al., 2020). It will be of interest to delineate the abnormalities in SCZ patient-specific dopaminergic neurons at the molecular and cellullar levels. Rakovic et al. generate a TH-mCherry iPSC reporter line by CRISPR/Cas9 technology to enrich the population of electrophysiologically mature TH^+^ dopaminergic neurons. This method can be applied to SCZ patient-specific iPSC lines for underpinning the dysfunction of dopaminergic neurons.

At the regional level, limbic regions such as the hippocampus and amygdala are known to play a role in working memory processes, but the relationship between structural changes in these regions and cognitive deficits in schizophrenia is complex and influenced by various factors, including the severity of the condition. Three articles in this Research Topic provide new insights into this issue. Cheng et al. suggest that the disrupted integration of the default mode network (DMN) contributes to working memory deficits in SCZ patients with severe attention problems. They use graph theory to examine the network topology of the brain during a working memory task and at rest in SCZ patients with different levels of attention deficit severity. The results show that patients with severe attention deficits have a higher normalized path length of the DMN compared to those with mild attention deficits and healthy controls, which are not sustained during rest. These findings might provide reliable biomarkers for attention deficits during working memory tasks for schizophrenia patients. Peterson et al. show that structural atrophy in the head and tail of the hippocampus and widespread amygdala positively correlated with the severity of symptoms and inversely with working memory performance in SCZ patients. They suggest that patients in different severity groups might form a spectrum of severity, as their working memory deficits and brain structural abnormalities follow similar patterns, but with varying degrees of severity. Delgado-Sallent et al. adopted a phencyclidine (PCP) mouse model to investigate the effects of NMDAR hypofunction on neural activities in the medial prefrontal cortex (mPFC) and the dorsal hippocampus (dHPC) during memory acquisition. They find that mice with subchronic PCP treatments exhibit impairments in short-term and long-term memory, which is associated with the disrupted mPFC-dHPC connectivity and the memory deficits are alleviated with Risperidone treatments by targeting this circuit. They suggest that this phenomenon might apply to SCZ patients with NMDAR hypofunction. An extracellular matrix protein Reelin is associated with SCZ in the human and mouse (Fatemi et al., 2001; Ishii et al., 2016; Negrón-Oyarzo et al., 2016). In this Research Topic, Pardo et al. report the effects of Reelin levels on adult brain's striatal structure and neuronal composition. They show that Reelin knockout mice (Cre fR/fR) from p45-60 onwards do not exhibit aberrant striatal structure and neuronal composition, whereas Reelin overexpressing (TgRln) mice display increases in the densities of striatal cholinergic interneurons and Parvalbumin interneurons in the ventral-medial striatum, dopaminergic projections in the ventral striatum, the number of dopaminergic synaptic boutons in the NAcc. They suggest those effects might play a counteracting role in the excitatory/inhibitory imbalance.

In closing, each SCZ study provides a piece of the puzzle. When many pieces of the puzzle fall into place, the pathophysiology of SCZ will be apparent. We hope that day is coming soon.

## Author contributions

All authors listed have made a substantial, direct, and intellectual contribution to the work and approved it for publication.
